# Novel 2-Benzylbenzimidazole Opioids: Emerging Drugs of Abuse and Pharmacological Considerations with Nitazene Analogs

**DOI:** 10.1007/s11916-026-01525-0

**Published:** 2026-07-22

**Authors:** Mary Kathryn Maxwell, Ahmed I. Anwar, Jamal Hasoon, Alan D. Kaye

**Affiliations:** 1 School of Medicine, Louisiana State University Health Sciences Center at Shreveport, Shreveport, LA 71103 USA; 2 Department of Anesthesiology, Critical Care, and Pain Medicine, The University of Texas Health Science Center at Houston, Houston, TX USA; 3 Department of Anesthesiology, Louisiana State University Health Sciences Center at Shreveport, Shreveport, LA 71103 USA

**Keywords:** Nitazenes, 2-Benzylbenzimidazole, Opioids, Synthetic opioids, Counterfeit drugs

## Abstract

**Purpose of Review:**

To summarize the developing evidence on novel 2-benzlbenzimidazole opioids (nitazene analogs), including pharmacology, potency, toxicology, management of overdose, and public health issues that are caused by this set of compounds as there is an increasing presence in illicit drug markets.

**Recent Findings:**

Nitazene analogs include those such as isotonitazene, metonitazene, protonitazene, and etonitazene are highly potent mu opioid receptor agonists and are increasingly detected in counterfeit and illicit pharmaceutical products with exposure in other substances including other drugs. Reports from multiple clinical, forensic, and public health investigations have all demonstrated that severe opioid toxicity can be characterized by profound respiratory depression, which is seen in nitazene compounds. Detection remains a central issue with this compound because these derivatives frequently are missed in routine toxicology screening.

**Summary:**

Nitazene analogs represent a new class of synthetic opioids that are associated with a high overdose risk related to high potency and increased prevalence worldwide. New techniques for overdose reversal include improvements in the pharmacology of opioid antagonists, prolonged and improved monitoring, and advances in life support inside and outside the hospital setting.

## Introduction

Over the past several decades, the opioid epidemic has evolved from predominantly heroin related misuse to increasing involvement of highly potent synthetic opioids that can be rapidly manufactured and widely distributed [1, 2]. While fentanyl and its analogs are causing a substantial proportion of overdose deaths, developing non-fentanyl synthetic opioids have begun to appear more frequently in illicit drug supplies and counterfeit pharmaceutical products [3]. Among these agents, nitazenes have appeared as a growing toxicologic and public health concern related to high potency, unpredictable formulation, and increasing prevenance in counterfeit tablets and polysubstance exposures [4].

Nitazenes belong to the 2-benzylbenzimidazole synthetic opioid class that was originally synthesized in the mid-20th century during efforts to develop potent analgesic agents [5]. Despite early pharmacologic interest, these compounds were never incorporated into routine clinical practice. In recent years, however, several nitazene analogs have re-emerged within illicit drug markets and have been implicated in overdose clusters worldwide, postmortem toxicology investigations, and counterfeit drug products commonly sold as oxycodone, benzodiazepines, or heroin [5].

Several nitazene analogs, including isotonitazene, metonitazene, protonitazene, etonitazene, and etodesnitazene, have demonstrated potent mu opioid receptor agonism in experimental and forensic studies [6]. Clinical concern surrounding these compounds stems primarily from side effect profiles including respiratory depression, particularly in individuals unaware of exposure or in cases involving co-ingestion of additional sedating substances [7]. The presence of nitazenes within counterfeit pills further increases overdose risk because users may unknowingly consume highly potent opioids without established opioid tolerance [7].

An additional challenge is that nitazenes are commonly not identified on routine hospital urine drug screening panels because they are structurally distinct from fentanyl and traditional tested opioid compounds [8]. Consequently, clinicians may encounter patients with classic opioid toxidromes despite initially negative toxicology results. This limitation has increased reliance on advanced analytical techniques, which include liquid chromatography tandem mass spectrometry for definitive identification and surveillance [9].

## Historical Background and Chemical Classifications

2-benzylbenzimidazole opioids were initially studied in the late 1950s as part of pharmaceutical research aimed at developing novel analgesic compounds with high opioid activity [10]. Nitazenes are synthetic opioids within the 2-benzylbenzimidazole family, representing a structurally distinct group compared with fentanyl, morphine, and other conventional opioid scaffolds. Chemically, nitazenes contain a shared benzimidazole core structure with a benzyl substituent and variable side chains which alter receptor activity, potency, and detectability [11]. Although several compounds in this class showed their potent opioid effects in early studies, nitazenes were never approved for routine human or veterinary medical use [7]. For decades, these compounds remained primarily within older medicinal chemistry and pharmacology literature, before they reappeared in contemporary illicit drug markets as non-fentanyl synthetic opioids [12].

The term “nitazene” is commonly used for 2-benzylbenzimidazole opioids that contain structural features associated with potent mu opioid receptor agonism [13]. Isotonitazene became one of the earliest modern nitazene analogs to attract major forensic attention after being identified in drug seizures and overdose investigations beginning around 2019 [10]. Since then, additional analogs including metonitazene, protonitazene, etonitazene, and etodesnitazene have been reported in forensic, toxicology, and drug checking settings [14]. Etonitazene is one of the historically recognized high potency members of this class and has served as an important reference compound for understanding newer analogs [13]. Isotonitazene is a closely related analog that gained attention because of its potent opioid activity and its involvement in modern overdose cases [10]. Metonitazene first appeared in mid-2020 in the recreational drug supply and has been increasing in death investigation casework since its emergence on the market [15]. Etodesnitazene is part of the broader benzimidazole opioid family and is often discussed with related compounds such as isonitazene and metonitazene in metabolism and toxicology studies [9]. The structural differences in these analogs matter clinically because small chemical modifications alter potency, metabolism, and the ability of routine toxicology screenings to detect exposure [14].

## Pharmacology

The 2-benzylbenzimidazole opioids primarily exert their effects through the agonism of the mu opioid receptor. Activation of this receptor inhibits ascending nociceptive signaling but also suppresses brainstem respiratory drive, which explains why severe opioid toxidromes can occur after a small exposure [6, 16]. In vitro studies show that many nitazene analogs demonstrate high mu opioid receptor affinity, high functional potency and agonist activity, with multiple agents that produce opioid receptor activation comparable to or greater than fentanyl [6]. Although nitazenes are often discussed as a class, minimal structural differences at the benzimidazole core, the N, N- dialkylaminoethyl side chain, as well as the para-substituted benzyl ring can profoundly alter receptor binding, potency, metabolism, and expected toxicity [17].

### Metonitazene

Metonitazene is a potent 2-benzylbenzimidazole opioid which acts as a mu opioid receptor agonist and has been identified in clinical and forensic toxicology settings [18]. Human metabolism work suggests that metonitazene undergoes several biotransformation pathways, including N-deethylation, O-demethylation, hydroxylation, nitro reduction, and glucuronidation, which is important for designing confirmatory toxicology assays [19]. Because metonitazene can produce opioid activation at low concentrations, unexpected exposure through counterfeit tablets or mixed drug supplies create an increased risk of someone having a overdose [6].

### Protonitazene

Protonitazene is a highly potent nitazene analog which also produces its main mechanism of action through being a mu opioid receptor agonist [18]. Experimental pharmacology has shown that protonitazene related compounds can display strong mu receptor activation, and newer pyrrolidino derivatives of protonitazene have been described along with emerging nitazene variants [13]. The high potency of protonitazene is clinically important because of its emergence in the drug market. Along with other nitazenes, protonitazene is expected to produce dose dependent opioid toxicity through central respiratory depression, sedation, and impaired protective airway reflexes [20].

### Isotonitazene

Isotonitazene was first detected in the recreational drug market in 2019, and since then, other synthetic opioids have increased in prevalence. Isotonitazene is an extremely effective nitazene analog with potency at the mu opioid receptor that exceeds that of fentanyl in terms of agonism [21]. Its pharmacologic risk is not limited to the parent compound because human metabolism studies have identified various metabolites, including products formed through N-dealkylation, O-dealkylation, hydroxylation, reduction, and conjugation pathways [22]. From a clinical standpoint, isotonitazene’s high receptor potency and potential for active metabolites help explain why intoxications present with profound central nervous system depression, miosis, and respiratory depression even when the exposure history may be unclear [6].

### Etonitazene

Etonitazene is a prototypical and historically important member of the nitazene class largely due to it being one of the most potent 2-benzylbenzimidazole opioids. It has been described as 1000-fold more potent than morphine is at the same receptor [23]. In vivo studies of etonitazene and related analogs demonstrate classic opioid-like pharmacodynamic effects, including antinociception, altered locomotor activity, and reduced body temperature in animal models [17]. These findings support the clinical expectation that etonitazene use can cause very high opioid toxicity, especially the common side effect of opioids being respiratory depression, even when the drug is present in very small amounts, highlighting its high potency [6].

Overall, 2-benzylbenzimidazole pharmacology is best understood as a combination of strong mu opioid receptor agonism, structural features that can markedly alter potency, and metabolic pathways that complicate detection [24]. These agents are especially dangerous in clinical practice due to their high potency, uncertain drug content, and incomplete detection on routine drug screening tests including urine screening, which can delay recognition of opioid poisoning [21].

## Potency

The remarkable potency of 2-benzylbenzimidazole opioids is a major factor contributing to their growing public health significance. Experimental pharmacology studies have demonstrated that nitazene analogs function as highly effective mu opioid receptor agonists, often producing receptor activation at concentrations substantially lower than those required for traditional opioids [6, 25]. Importantly, potency estimates vary according to the experimental endpoint evaluated, including receptor binding affinity, intracellular signaling assays, antinociceptive testing, and behavioral models. Consequently, potency rankings should be interpreted as approximations rather than absolute values [18]. Nevertheless, available pharmacologic and forensic evidence consistently places etonitazene among the most potent members of the nitazene class, while isotonitazene and protonitazene demonstrate potency exceeding that of fentanyl in many experimental systems [4, 26].

Metonitazene appears somewhat less potent than isotonitazene and etonitazene but remains capable of producing profound opioid toxicity at very low doses [18]. The extreme potency of these compounds is clinically important because even minor variations in drug concentration, tablet formulation, or user tolerance may substantially increase the apparent risk of respiratory depression and overdose causing death [27]. Furthermore, recent mechanistic investigations suggest that certain nitazenes exhibit prolonged µ-opioid receptor occupancy and slow receptor dissociation kinetics, properties that may contribute to severe toxicity and complicate reversal during overdose events [25]. These findings highlight why nitazenes continue to present significant challenges for clinicians, toxicologists, and public health agencies despite their relatively recent emergence in illicit drug markets [21]. A summary of the potency of these Nitazenes is found in Table 1.

**Table 1:** Comparison of Heroin Potency to Selected Nitazene Opioids

Drug	Relative Potency to Heroin
Heroin	1
Fentanyl	50
Metonitazene	50
Protonitazene	100
Isotonitazene	250
Etonitazene	500

Table adapted from [4, 28]

## Clinical Toxicology

Nitazene use commonly presents with a similar toxic side effect profile as other opioids, including sedation and hypoventilation, with the overdose sometimes progressing to severe poisoning requiring clinical intervention [29]. Clinical nitazene toxicity is characterized by profound CNS and respiratory depression, and some presentations range from somnolence to respiratory failure requiring mechanical ventilation [30]. Reports of intoxication describe progression to unconsciousness and severe CNS depression and there are some cases where the patients are found unresponsive after being exposed to nitazene [31]. Across different case reports, nitazene toxicity is characterized by apneic episodes and hypoventilation, including respiratory compromise requiring manual ventilation [29–31]. There have been cases of brain death following nitazene-induced respiratory failure have been reported, demonstrating the extreme toxicity of this drug class [21, 30]. Clinical management requires opioid reversal with naloxone which has shown to improve mental status and reverse sedation in multiple exposure cases [30, 31]. Real world exposure demonstrates that nitazene intoxication may occur through nontraditional routes, such as vaping and contaminated drug products, which complicates the clinical presentation of the patient who is experiencing the overdose [32].

### Overdose Manifestations

Clinical cases also document cardiac arrest secondary to hypoxemia, which reflects downstream consequences of opioid induced respiratory failure [29, 30]. Fatal outcomes have also been reported, including death and brain death, which demonstrates the high level of lethality of these drugs [21, 30, 31]. In severe cases, patients require intubation and mechanical ventilation. Severe toxicity has also been linked to vaping associated use of nitazene [21, 31].

### Comparative Toxicity Across Analogs

Nitazene analogs such as isotonitazene, metonitazene, are structurally related compounds with high mu opioid receptor agonist activity, and similar opioid toxidromes, which highlights the shared pharmacological mechanisms across this class [30, 32, 33]. In vivo pharmacological evaluations show that isotonitazene produces increased analgesic potency compared to fentanyl or morphine [33]. Collectively, these findings demonstrate that nitazene analogs share potent µ-opioid receptor agonist activity and produce similar opioid toxidromes despite structural differences among individual compounds.

## Overdose Management

The rise of nitazenes within illicit drug markets has introduced a new generation of highly potent synthetic opioids associated with significant toxicologic and public health concerns [34]. Recent articles have indicated that some nitazene analogs may exhibit receptor affinity and effectiveness that exceed both fentanyl and morphine, which contributes to its high overdose risk and profile [18]. Clinical manifestations of nitazene toxicity are similar to those of other opioids and these symptoms consist of respiratory depression, hypoventilation, and decreased levels of arousal [29, 34]. Nitazene-associated overdoses and fatalities are increasingly reported across North America and Europe, reflecting the growing presence of these substances in illicit drug markets [34]. Unintentional exposure is common because nitazenes are frequently encountered in counterfeit or adulterated products marketed as other substances [29]. Emergency department data suggest that overdoses including new potent opioids can be associated with the greater naloxone requirements than fentanyl overdose, supporting that nitazenes exhibit a very high clinical potency [35].

### Reversal Agents

Naloxone remains the first-line treatment for nitazene overdose and also continues to be effective in reversing the opioid-induced respiratory depression caused by nitazene exposure [18, 29]. Nitazene’s high potency and activation at the mu opioid receptor allows for naloxone reversal through competitive antagonism at this exact receptor [36]. In a case series of laboratory confirmed nitazene poisoning, naloxone successfully reversed toxicity, and repeat naloxone administration was required in approximately 45% of patients receiving naloxone, which highlights the extreme potency of this drug class, and the need for ongoing reassessment after initial reversal [29]. Experimental data suggests that nitazene analogs dissociate slowly from the mu opioid receptor and are more resistant to naloxone antagonism than the rapidly dissociating opioids, which suggest that an increased dose may be necessary in such overdoses [25]. Some patients require prolonged naloxone infusion to improve to adequate respiratory function due to the high potency of this drug [18]. Early naloxone administration remains critical because reversal of opioid respiratory depression reduces the mortality risk of these drugs [36].

Nalmefene is a longer acting mu opioid receptor antagonist and was approved by the U.S. FDA in 2023 as a intranasal formulation indicated for the reversal of opioid overdose [37]. Nalmefene has a longer elimination half-life than naloxone (approximately 11.4 h), which can reduce the risk of recurrent opioid toxicity after initial reversal. Concerns still remain about its prolonged duration of action as resulting in longer-lasting opioid withdrawal symptoms than naloxone however. Naloxone is still the standard of care, and additional evidence is needed before a longer acting antagonist such as nalmefene can be recommended to replace naloxone [38, 39].

### Airway and Critical Care Management

Management of nitazene overdose, and its analogs, which share similar toxidromes, follow a standard opioid poisoning protocol with immediate assessment of the airway, breathing and circulation [18]. The primary goal is restoration of ventilation and oxygenation rather than complete reversal of sedation [36, 40]. Supplemental oxygen is administered as indicated, and assisted ventilation should be provided in patients who do not have enough respiratory effort [18, 36]. Patients with severe respiratory depression, hypoxemia, or failure to respond to naloxone may require endotracheal intubation and mechanical ventilation [29]. In an Australian nitazene case series, severe poisoning resulting in hypoxemia or cardiac arrest required intubation, demonstrating the need for more care depending on the patient severity of overdose [29]. Intensive care unit admission may also be necessary for some patients who have prolonged respiratory depression or other overdose symptoms [18]. Continuous monitoring is also recommended following reversal because of recurrent opioid toxicity after the initial improvement [18, 29, 36]. Polysubstance exposure is also a common cause of nitazene overdose and may complicate the ability to diagnose the overdose, clinical presentation of the overdose, and treatment decisions [18]. These findings emphasize the importance of continuous monitoring following apparent improvement in patient arousal and respiratory function.

## Public Health Challenges

A major challenge in detecting nitazene exposure is that these compounds are frequently missed by routine hospital immunoassay drug screening panels [41]. Nitazenes are structurally distinct from morphine, heroin, and fentanyl, resulting in minimal cross-reactivity with antibodies used in the routine immunoassay drug screens and increasing the likelihood of there being a false-negative result. Consequently, routine screening methods are often insufficient for reliably identifying nitazene exposure in different settings including clinical. Identification relies on advanced analytical techniques such as LC-MS/MS, LC-QTOF-MS, or LC-HRMS, which are required for accurate detection of the analogs in biological and seized material samples [42]. Recent work on nitazene immunoassays test strips show partial effectiveness as a rapid screening tools, with these strips detecting 28 of 36 nitazene compounds [43]. However, performance limitations are observed, with false negatives, and false positives, both being linked to high caffeine concentrations [43]. A concern is raised regarding reliability in reduction settings, with further evaluation of immunoassays showing that detection varies significantly across the structural subclasses of nitazene. Matrix effects, composition, and environmental conditions also impair test performance. Analysis of nitazene tools confirm that while immunoassays may function as a screening method, they will not replace confirmatory mass spectrometry at the moment because of the inconsistent sensitivity and specificity [14].

### Counterfeit Drugs

Nitazenes are found in counterfeit and misrepresented drug products, which adds to its high-risk profile, as it is found in products sold as heroin and false prescriptions such as other opioids or benzodiazepines [44]. This creates a major risk of unintentional exposure, especially among individuals unaware of consuming synthetic opioids [44]. The issue is compounded by the production of counterfeit pills, which are almost indistinguishable from legitimate sources. These pills broaden the exposure profile to synthetic opioids beyond traditional drug using populations, which includes adolescents and recreational users who are under the impression they are consuming legitimate medications [45]. As a result, individuals who would not intentionally use synthetic opioids with such high potency, may still be exposed to a highly potent nitazene. The deception is inherent in counterfeit pharmaceuticals, and it increases the overdose risk, since users cannot predict potency or composition. Nitazenes may be present alone or in combination with other novel psychoactive substances, which further causes more toxicity [41, 44]. This shift toward disguised pharmaceutical products represents a major public health concern that expands the population at risk that would not have otherwise be at risk [45, 46].

### Public Health Implications

The surveillance of nitazenes is complicated by analog development and arrival of new variants that outpace the detection systems [47]. As a result, forensic and public health monitoring systems lag behind the market evolution, which causes delayed identification and further spread of these illicit drugs [47]. Limited forensic laboratory capacity may further contribute to underrecognition of nitazene involvement in overdose deaths [42]. Large forensic datasets do indicate significant growth and prevalence. Nearly 7,000 nitazene submissions were reported in the U.S. National Forensic Laboratory Information System (NFLIS) between 2019 and 2025, accentuating their expanding reach and presence [47]. Wastewater based epidemiology surveillance also is evolving and provides insight into population level exposure. Detection of nitazene analogs in wastewater samples in multiple countries demonstrate the global distribution of nitazenes [28]. Detection remains geographically inconsistent which indicates uneven coverage. Public health wise, nitazenes are increasingly associated with overdose deaths across multiple regions, including the United Kingdom, Norway, Australia, and the United States [45]. The high potency, combined with unintentional exposure and a delay in detection contribute to its high mortality risk [45]. Public health recommendations are emphasizing expanded surveillance systems, increased naloxone access, improved clinician awareness, and targeted education [41, 45, 48].

## Conclusion

2-Benzylbenzimidazole opioids (nitazenes) have rapidly emerged as an important class of synthetic opioids within the evolving illicit drug market. Although originally synthesized decades ago, recent re-emergence has been accompanied by increasing reports of overdose, fatalities, and detection in counterfeit pharmaceutical products. Their potent µ-opioid receptor agonism, variable pharmacologic profiles, and ability to produce profound respiratory depression contribute to a substantial risk of morbidity and mortality. In this regard, healthcare professionals need to understand the growing use of these compounds and potential risks.

The growing prevalence of nitazenes presents significant challenges for clinicians, toxicologists, and public health officials. Routine toxicology screening methods often fail to detect these compounds, complicating diagnosis and surveillance efforts. While naloxone remains an effective treatment for opioid toxicity, the high potency and prolonged receptor interactions of certain nitazene analogs may necessitate repeated dosing, continuous monitoring, and advanced airway management. Continued surveillance improved analytical detection methods, expanded harm reduction initiatives, and increased clinician awareness will be essential to addressing the evolving threat posed by these emerging synthetic opioids.

## Key References

- Schwarz ES, Dicker F, Lothet E, Spungen H, Levine M. Nitazenes: An Old Drug Class Causing New Problems. Missouri Medicine. 2025;122(4): 329–333.
  - ○ Supports the public health context and potency comparison for nitazenes
- Kozell LB, Eshleman AJ, Wolfrum KM, Swanson TL, Bloom SH, Benware S, et al. Pharmacologic Characterization of Substituted Nitazenes at μ, κ, and Δ Opioid Receptors Suggests High Potential for Toxicity. The Journal of Pharmacology and Experimental Therapeutics. 2024;389(2): 219–228. https://doi.org/10.1124/jpet.123.002052.
  - ○ Helps describe the fundamental pharmacology of nitazenes, its agonism, potency, and mechanistic basis for toxicity
- Stangeland M, Dale O, Skulberg AK. Nitazenes: review of comparative pharmacology and antagonist action. Clinical Toxicology. 2025;63(6): 393–406. https://doi.org/10.1080/15563650.2025.2504133.
  - ○ Connects clinical relevance to toxicology, helping explain real world implications of nitazenes

## Data Availability

No datasets were generated or analysed during the current study.
